# Epoxyorganosilane Finishing Compositions for Fibrous Fillers of Thermosetting and Thermoplastic Binders

**DOI:** 10.3390/polym14010059

**Published:** 2021-12-24

**Authors:** Alexey V. Shapagin, Natalia A. Gladkikh, Arkadiy A. Poteryaev, Valentina Yu. Stepanenko, Uliana V. Nikulova, Ramil R. Khasbiullin

**Affiliations:** Frumkin Institute of Physical Chemistry and Electrochemistry, Russian Academy of Sciences, Leninsky Prospect 31-4, 119071 Moscow, Russia; fuchsia32@bk.ru (N.A.G.); apoteryaev@gmail.com (A.A.P.); 4niko7@list.ru (V.Y.S.); ulianan@rambler.ru (U.V.N.); khasbiullin@techno-poisk.ru (R.R.K.)

**Keywords:** organosilane, epoxy resin, epoxyorganosilane system, fiber finish, finishes

## Abstract

The development of universal finishing compositions for fibers of various natures is an urgent task for polymer composite materials science. The developed finishes can be used for the fiber reinforcement of polymer matrices with a wide range of surface free energy characteristics. Epoxy systems modified with diaminesilane in a wide concentration range were examined by optical interferometry, FTIR spectroscopy, DSC and the sessile drop technique. It was shown that the partial curing of epoxy resin by diaminesilane at room temperature under an inert atmosphere, followed by contact with air, leads to a significant increase of the surface free energy of the system. Varying the concentration of diaminesilane allows us to effectively regulate the surface free energy of the composition. This makes it possible to use fibers finished with epoxyaminosilane compositions in composite materials based on a various thermosetting and thermoplastic binders with a surface tension of up to 75 mJ/m^2^.

## 1. Introduction

One of the modern problems of polymer materials science associated with the improvement of the physical and mechanical properties of composite materials is not only the development of new polymer binders and reinforcing fibers but also the obtainment of good adhesion strength at the interface due to the formation of physical and chemical bonds, which ensures the cooperative functioning of all of the structural elements of the composite material as a whole.

The fibers used to obtain fibrous composite materials (carbon, aramid, glass and others) have high values of tensile strength, a modulus of elasticity, heat resistance and low density [1,2]. These properties make fibers indispensable components in the creation of structural composite materials based on thermoplastic and thermoset binders, which are widely used in various industries: space, aviation, shipbuilding, automotive, and many others [3,4,5,6,7]. In order to impart the high technological characteristics of the fibers, and to exclude damage when they are in contact with the winding rollers of weaving textile equipment, a thin finishing layer is applied to their surface. In addition to solving the technological problem, the finishing layer must have strong adhesion to the fiber, and must ensure the fiber wetting by the binder. In order to increase the surface energy of the fiber in the absence of a finishing coating, the methods of physicochemical modification are often used [1,8]: plasma and ultrasound treatment, gamma radiation, ultraviolet radiation, and chemical and electrochemical treatment. Such processing methods allow us to increase the amount of oxygen-containing groups on the fiber surface, which leads to the increase of the polar component of the surface energy. In this way, the affinity of the components at the interface rises, and good surface wetting with substances with lower surface tension is achieved [9]. A major disadvantage of these methods is a significant decrease in the physical and mechanical characteristics of the fibers. In addition, the usage of different types of binders entails new requirements for the surface energy of the fiber. As mentioned above, good surface wettability will be ensured if the surface tension of the liquid is close to or less than the value of the surface energy of a solid [10]. Thus, in the development of modern fibrous polymer composite materials, it is important to have finishing compositions that not only ensure the good wetting effect of the surface of the fiber but also provide the affinity at the treated fiber–binder interface. Recently, along with thermoset binders, various thermoplastic, thermoset matrices modified by thermoplastics [2,11,12,13,14,15,16,17,18] and fibrous composite materials [4,5,6,19,20,21,22] have been widely used. Thus, the problem of the modification of the existing finishing materials with the aim of directly changing the surface energy characteristics is urgent. The indisputable advantage of fiber finishing as a technique of surface treatment is the simplicity and low cost of its usage in industrial production. In addition, this technique allows us to achieve high adhesion strength at the phase interface without losing the physical and mechanical characteristics of the fiber [23,24].

Epoxy oligomers are used as chemical finishes for fibers. Fibers treated with epoxides are often used for epoxy matrices’ reinforcement. An analysis of the literature showed that epoxy oligomers modified with organosilanes and mixtures of organosilanes with thermoplastics are used as finishing compositions [17,25,26,27,28,29]. Organosilanes are artificial organosilicon compounds obtained from silicon dioxide [30,31]. The chemical structure of organosilanes is R_(4-n)_–Si–(R’X)_n_, where n = 1 or 2; X is an organofunctional group such as vinyl, amino, methacryl, or epoxy, etc. [32]; R’ is an alkyl chain, which connects a silicon atom and the organofunctional group [33]; R is a hydrolysable alkoxy group containing f.e. methoxy (–OCH_3_), ethoxy (–OC_2_H_5_), or acetoxy (–OCOCH_3_) [30,34]. As a result of the condensation reaction, polysiloxane compounds with thermally stable siloxane bonds (≡Si–O–Si≡) are formed. The presence of a polar substituent of the hydrocarbon fragment bonded to a silicon atom leads to an increase in the polarity of the polymer molecule and, as a consequence, to an increase in the adhesion characteristics, mechanical strength, and other properties [34,35,36].

Organosilanes with functional NH groups can chemically interact with the epoxy group of the epoxy oligomer [26], forming a cross-linked structure. The hydrolysable alkoxy groups increase the polar component of the free surface energy of the modified epoxide. Thus, as a result of the processing of a fiber with epoxyaminosilane is that its good wettability with an epoxy binder should be expected, and high adhesion strength between the finished fiber and the matrix of the composite can be achieved [17,37,38,39].

According to [40,41,42,43,44], the modification of epoxy resin with organosilanes is mostly applied to improve the elasticity and corrosion resistance of the coating. Investigations dedicated to the modification of epoxy resin with organosilanes to use the compositions as fiber finishes are rare, and are of significant interest.

The aims of the current research are to study the physicochemical properties of the epoxy oligomer–amine-containing organosilane system, and to recommend various compositions for fiber finishing. Fibers treated with such compositions can be used to obtain composite materials based on thermosetting and thermoplastic matrices with various surface free energies.

## 2. Materials and Methods

### 2.1. Used Materials

The components of the investigated organosilane epoxy system were:

- epoxy resin ED–20 (ER), С_21_Н_24_О_4_ (Propitay, Moscow, Russia);
- aminoethylaminopropyltrimethoxysilane–diaminesilane (DAS), С_8_Н_22_N_2_O_3_Si (Arsenal-kama, Perm, Russia).

DAS was used as a curing agent for ER. Curing occurs due to the interaction of epoxy groups with the amino groups of DAS. Another purpose of using DAS was to make it possible to regulate in a wide range the surface free energy of the composition.

The objects of study were mixtures of ER with DAS at concentrations ranging from 5 to 45 wt%, with a 5 wt% step (Table 1).

Pre-curing was carried out at room temperature in an argon atmosphere for 24 h. An inert atmosphere during the pre-curing process is necessary to avoid the hydrolysis of the DAS during the composition preparation. Preliminary studies have shown that the curing of the epoxy–DAS systems under air conditions leads to the formation of a heterogeneous phase structure.

The post-curing of the epoxyorganosilane compositions was carried out at 160 °C for 2 h in an air atmosphere.

### 2.2. Optical Interferometry

In order to study the compatibility of components in the entire concentration range, we used the method of optical interferometry. All of the measurements were performed on an ODA–2 diffusiometer (IPCE RAS, Moscow, Russia) [13,45,46]. A helium–neon laser (λ = 632.8 nm) was used as a source of monochromatic light with a parallel beam. The optical wedge was about 100 µm thick.

A drop of ER was placed in a diffusion cell between quartz glasses with the inner sides covered with a semitransparent layer of NiCr alloy. A small angle ≤2° was established between the glasses, which is necessary for the appearance of an interference pattern. The diffusion cell was thermostated for 20 min at a temperature of 25 ± 0.5 °C, and DAS was injected between the glasses. 

The mutual solubility of the components can be defined using the change of the refractive indexes in the diffusion area. The interference fringes’ curvature and the presence/absence of phase boundaries in the diffusion zone were analyzed. Each fringe in the diffusion area is isoconcentrational (corresponds to a certain concentration). The position of the isoconcentration fringes was used to construct a diffusion profile, which made it possible to draw conclusions on the compatibility of the components at any concentration. Kinetic studies of the concentration profile made it possible to calculate the diffusion coefficients, and to record the appearance of phase decomposition.

### 2.3. Attenuated Total Reflectance Fourier Transform Infrared Spectroscopy (ATR–FTIR)

The FTIR spectra of the ER, DAS and their cured mixtures (see Section 2.1) were obtained using a Nicolet iN10 FTIR spectrometer (Thermo Fisher Scientific, Waltham, MA, USA) in the spectral range 4000–675 cm^−1^. The spectra were taken on germanium crystal in ATR mode as the average of 128 scans at a resolution of 4 cm^−1^. The processing was carried out using the Omnic 9 software (Thermo Fisher Scientific, Waltham, MA, USA).

### 2.4. Differential Scanning Calorimetry (DSC)

In order to identify the changes in the temperatures of the phase transitions of epoxyorganosilane systems during curing, DSC was used. The DSC thermograms were measured on Netzsch DSC 204F1 (Netzsch, Selb, Germany). The initial substances and the bicomponent mixtures cured at 25 °C were cooled to −50 °C and then heated to 100 °C at a heat rate of 20 K min^−1^. Then, the pre-cured samples were post-cured at a temperature of 160 °C for 2 h. In order to identify the changes in the chemical structure of the post-cured samples, DSC measurements were carried out in the temperature range from −50 to 200 °C at a heat rate of 20 K min^−1^. 

### 2.5. Surface Free Energy Definition

In order to determine the surface free energy of epoxyorganosilane systems cured at various temperatures, the sessile drop technique was used. The technique is based on the analysis of the shape of droplets of test liquids on the sample surface. Contact angle measurements were carried out at room temperature using an EasyDrop Standard tensiometer (KRUSS, Hamburg, Germany). A set of test liquids (Table 2) with a wide range of surface tension values was used. The horizontal optical microscope camera of the tensiometer recorded the process of a droplet spreading over the surface to the equilibrium state. The contact angle θ between the sample and the tangent to the droplet surface was measured.

Using the Owens-Wendt Equation (1), the surface energies (γ) and their polar (γ^Р^) and dispersive (γ^D^) components were calculated.

(1) $$\frac{\gamma_{L}\times (1+\cos\theta )}{2\sqrt{\gamma_{L}^{d}}}=\sqrt{\gamma_{S}^{\rho }}\times \sqrt{\frac{\gamma_{L}^{\rho }}{\gamma_{L}^{d}}}+\sqrt{\gamma_{S}^{d}}$$

where $\gamma_{L}$, $\gamma_{L}^{d}$ and $\gamma_{L}^{\rho }$ are the total, dispersive and polar components of the test fluid surface free energy, respectively; $\gamma_{S}^{d}$ and $\gamma_{S}^{\rho }$ are the dispersive and polar components of the solid surface free energy; and θ is the contact angle.

## 3. Results and Discussion 

### 3.1. Investigation of the Finishing Compositions 

While developing the fiber finishing compositions suitable for use with various binders, in our opinion, two main problems should be solved. The first is to ensure good wetting at the fiber–finish and finish–binder interfaces. This can be achieved if the surface tension of the liquid phase is close to or less than the surface free energy of the solid in contact with it (the fiber finish or fiber). The second is obtaining the system in which a predicted change in energy characteristics occurs by changing the concentration of the modifying component.

**Figure 1:**
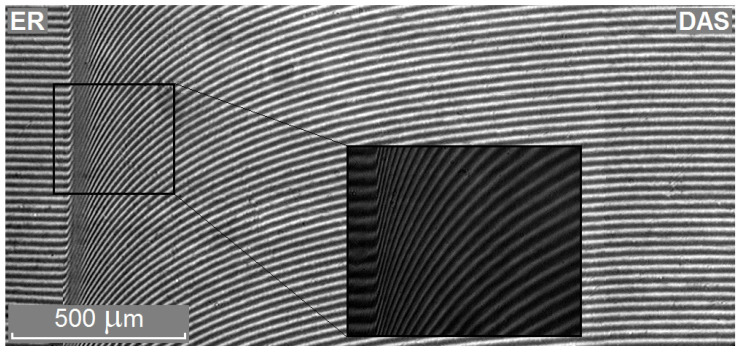
Typical interferograms of the interdiffusion zones of the epoxyorganosilane system at 25 °C.

It is known that for fully compatible systems, the physicochemical properties vary according to the additive law. Therefore, at the beginning of the work, special attention to the checking of the compatibility and mutual solubility of the initial components of the epoxyorganosilane system was paid. Figure 1 shows a typical interferogram of the interdiffusion zone of the semi-infinite media of the epoxy oligomer (left) and DAS (right) in 25 °C. 

**Figure 2:**
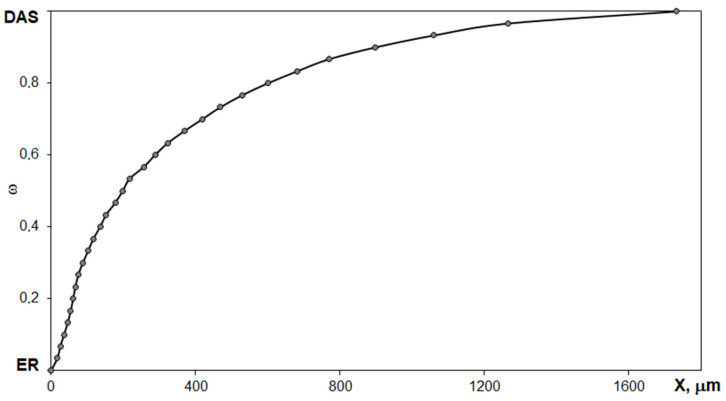
The diffusion profile of the epoxyorganosilane system at 25 °C.

The S-shaped curvature of the diffusion profile and the absence of a phase boundary indicate a continuous change in the refractive index with the concentration of the components. Figure 2 shows the diffusion profile of the epoxyorganosilane system at 25 °C.

**Figure 3:**
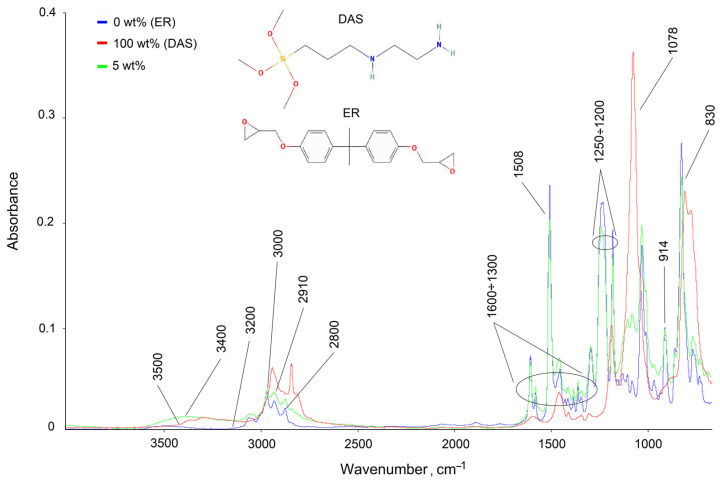
FTIR spectrum of 0 wt% (ER), 100 wt% (DAS), and their epoxyorganosilane system.

The continuity of the diffusion profile indicates the compatibility of the studied components in the entire concentration range. During the chemical reaction of curing at 25 °C, the shape of the diffusion profile did not change. A decrease in the expansion rate of the diffusion zone due to a decrease in the interdiffusion coefficients was recorded. In all probability, the deceleration of the mass transfer process is due to the formation of a spatial network as a result of a chemical interaction of the epoxy and amine groups of the initial components. In order to determine the chemical transformations in the epoxyorganosilane systems, FTIR spectroscopy in spectral range 4000–675 cm^−1^ was carried out (Figure 3).

The spectrum of 0 wt% (ER—blue curve) correlates well with the reference data [28,41], and the spectrum of 100 wt% (DAS—red curve) corresponds to the vibrations of the bonds of its chemical structure. The presence of characteristic bands of weak stretching vibrations of the hydroxyl group of ER in the region of 3500 cm^−^^1^ and stretching vibrations of amine groups of diaminsilane (3400–3200 cm^−^^1^) are clearly seen. The stretching vibrations of the =C–H, –CH_2_– and –CH_3_ groups of epoxy resin and organosilane in the range from 2800 to 3000 cm^−^^1^ were also recorded. Bands in the range of 1600 to 1300 cm^−^^1^ are related to different kinds of vibrations of aromatic rings of ER. The absorption band at 914 and 1232 cm^−^^1^ was assigned to the C–O and C–O–C stretching vibrations of the epoxide group. The broad band at 1078 cm^−^^1^ corresponds to the stretching vibrations of the Si–O–C of DAS.

The spectrum of the epoxyorganosilane system 5 wt% (green curve) is similar to the spectrum of the epoxy oligomer, but already has a number of significant differences confirming the crosslinking of the epoxy resin. First, the number of C–O–C bonds (1232 cm^−^^1^) and epoxy groups (1232 cm^−^^1^, 914 cm^−^^1^, 830 cm^−^^1^) decreases. Secondly, as a result of the epoxy ring opening, the intensity of the stretching vibrations of the hydroxyl bands increases. It should be noted that there are signs of DAS hydrolysis. The emergence of absorption bands corresponding to Si–OH (1040–1020 cm^−1^ and 990–945 cm^−1^) confirms that the –O–CH_3_ groups of DAS are modified. The absorption band in the region 1090–1010 cm^−1^ (Si–O–Si) is a result of linkage formation.

**Figure 4:**
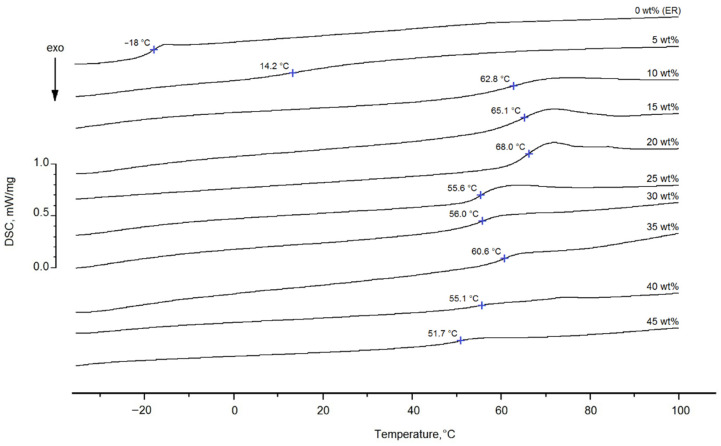
The DSC thermograms of the epoxyorganosilane system with different concentrations of diaminesilane, 5–45 wt%, cured at 25 °C.

In order to determine the degree of conversion of the epoxy groups for the epoxyorganosilane system with different concentrations of DAS, thermochemical investigations were carried out. The DSC thermograms of the investigated systems cured at 25 °C are shown in Figure 4.

It was found that the pre-curing of the epoxyorganosilane system of 5 wt% at 25 °C increases the glass transition temperature (T_g_) from −18 to 14 °C. A further increase of the DAS content in the epoxyorganosilane system to 10 wt% leads to an increase of the T_g_ to 63 °C. It was found that the T_g_ of pre-cured compositions with higher organosilane content (from 15 to 45 wt%) are in range of 52–68 °C. Thus, the varying of the DAS concentration in the range of 10–45 wt% does not lead to a noticeable change of the T_g_ or a change in the crosslink density, consequently. This can be explained by the fact that the T_g_ of the system during the curing reaction slowly increases, and when T_g_ reaches the reaction conditions (25 °C), the curing reaction rate slows down due to a decrease in macromolecular mobility. It is important that the deformation properties of such systems, which have not reached the gel point, meet the technological requirements for their use as compositions for a finishing fiber.

As mentioned earlier, for the effective use of polymer–polymer systems as finishes for various fibers which can be used for various composite materials reinforcement, it is necessary to be able to regulate the surface free energy characteristics in a wide range. The obtained dependences are compared with the energy characteristics of the epoxy oligomer, the initial DAS, and DAS cured as a result of the hydrolysis reaction in an air atmosphere (Table 3).

The surface tension of ER and DAS in a liquid physical state was obtained by the sessile drop method and calculated using Equation (1). Test surfaces with different surface energy characteristics are listed in Table 4.

The determination of the energy characteristics of the epoxyorganosilane system at 5% was hindered due to the highly inhomogeneous molecular structure at the initial phase as a result of microgel structure formation (T_g_ < T_experiment_). It was found that, for the system at 10 wt%, the surface energy of the cured composition increased from 56 to 75 mJ/m^2^ due to increase of polar components of the surface energy. It should be noted that the value of the surface energy and its polar and dispersed components of the system at 10 wt% are comparable with the values of the diaminesilane cured as a result of the hydrolysis reaction. The high value of the polar component of the surface free energy is due to the migration of diaminesilane into the near-surface layers, and the formation of hydroxyl groups after the system enters the air atmosphere. This was confirmed by the FTIR spectrum. The absorption in the region of hydroxyl groups’ stretching vibrations is comparable for diaminesilane and epoxy oligomer for the system at 5 wt% (Figure 3). 

**Figure 5:**
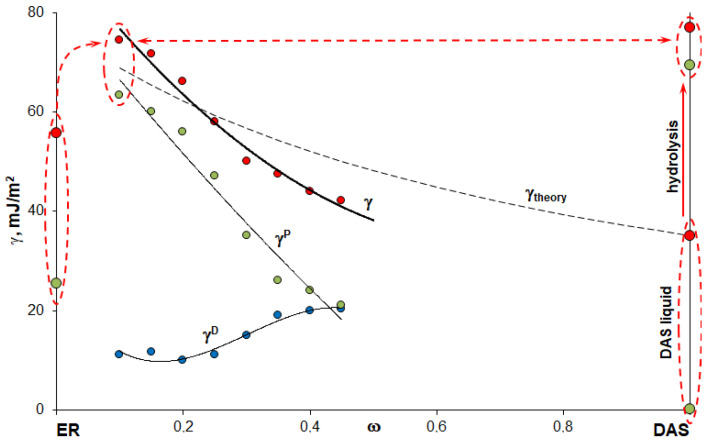
Concentration dependences of the surface free energy (red points), and its polar (green points) and dispersion (blue points) components. The dashed line indicates the theoretical dependence of the surface free energy according to Equation (2).

An increase of the DAS concentration in the epoxyorganosilane system leads to a decrease in the polar component of the surface free energy and, as a result, in the total surface free energy of the cured systems. One of the reasons for such a change in the energy characteristics is DAS molecules’ mobility reduction is the chemical network formation. As a result, the concentration of DAS at the near-surface and the density of the hydroxyl groups on the surface decreases. Another reason is the additive change of the properties of the homogeneous epoxyorganosilane system (Figure 2, Equation (2)). The concentration dependence of the obtained values of the total surface free energy of the investigated systems is similar to the calculated curve (Figure 5, dashed curve). It can be seen that the dispersive component of the surface free energy increases as a result of the formation of dense microgel structures.

(2) $$\frac{1}{\gamma_{theory}}=\frac{\omega_{ER}}{\gamma_{L}^{ER}}+\frac{\omega_{DAS}}{\gamma_{L}^{DAS}}$$

where $\omega_{ER}$ and $\omega_{DAS}$ are the ER and DAS concentrations, $\gamma_{L}^{ER}$ and $\gamma_{L}^{DAS}$ are the surface free energies of the ER and DAS respectively, and $\gamma_{theory}$ is the surface free energy of the composition.

Thus, by curing the epoxyorganosilane system in an inert atmosphere at 25 °C, partially-cured stable compositions with the T_g_ in the range of 52–68 °C can be obtained. The surface energy of the composition additively changes from 75 to 42 mJ/m^2^ with an increase in the concentration of DAS from 10 to 45 wt%. The residual content of the reactive groups of the epoxy resin can facilitate the formation of a stronger adhesive contact at the interface between the phases of the finish-matrix of the fibrous composite material.

**Figure 6:**
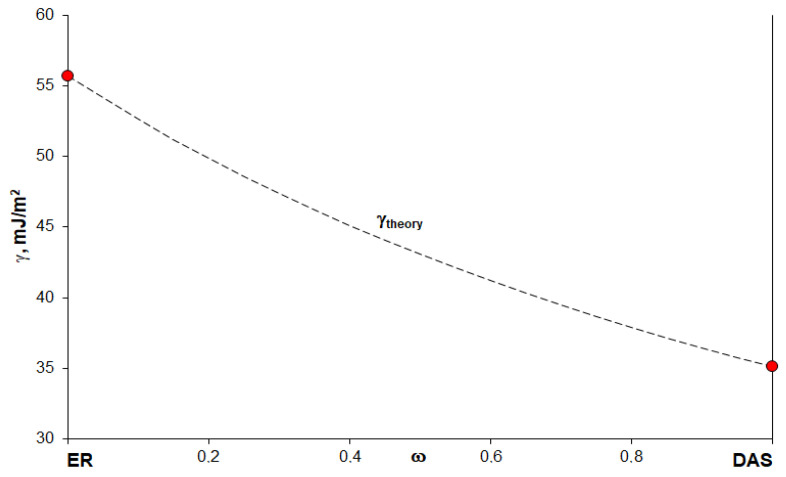
Theoretical dependence of the surface free energy on the concentration of the initial uncured components in the epoxyorganosilane system.

It is known that, in order to ensure good wetting, it is necessary to comply with the condition when the surface tension of the binder is lower than the surface energy of the wetted surface. The wide range of values of the surface energy of the investigated compositions makes it possible to use them for the finishing of the fibers, which, subsequently, can be used for the fabrication of composite materials based on binders with surface tensions up to 75 mJ/m^2^. It is important that the surface tensions of the original (uncured) homogeneous epoxyorganosilane system are rather low (Figure 6).

This makes it possible to ensure good wetting during the finishing process of different types of fibers. In some cases, in order to ensure better wetting, it is recommended to increase the surface energy of the fibers, for example by oxygen discharge plasma treatment [2,3].

### 3.2. Post-Curing of the Finishing Compositions

**Figure 7:**
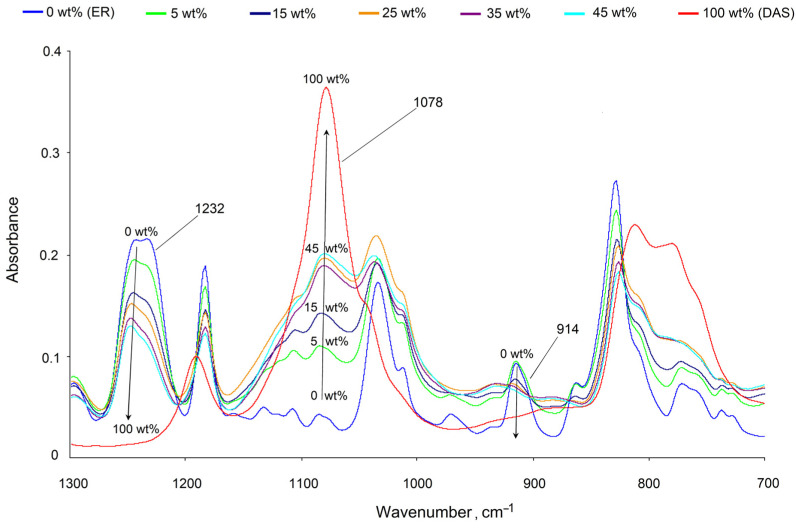
FTIR spectrum of 0 wt% (ER), 100 wt% (DAS), and their epoxyorganosilane systems with different concentration of DAS. The arrows show the increased concentration of DAS.

At the second stage of the current research, additional curing of the investigated compositions at 160 °C for 2 h was carried out. The changes in the molecular structure of the systems are shown in Figure 7.

**Figure 8:**
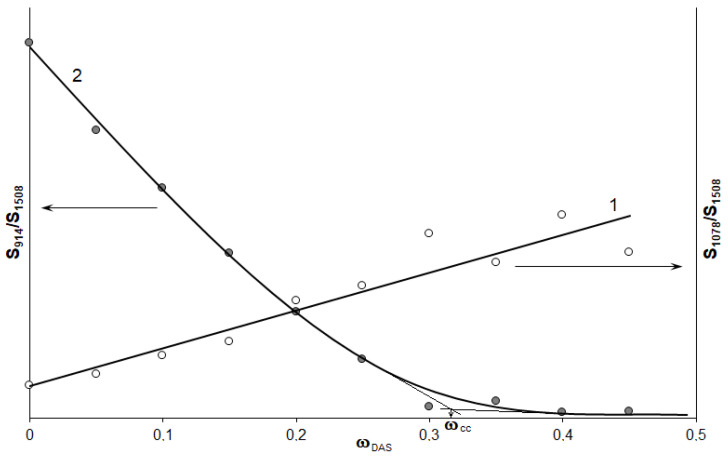
Concentration dependences of the ratio of the 1078 cm^−^^1^ (1) and 914 cm^−^^1^ (2) peak areas to the 1508 cm^−^^1^ peak area.

The FTIR spectra of various mixtures show changes in the range of 1300 to 700 cm^−1^. The intensity of the 1078 cm^−1^ band sharply increases with an increase of the concentration of DAS. At the same time, a decrease in the intensities of the 914 cm^−1^ and 1232 cm^−1^ bands is observed. The absorption peak in the 914 cm^−1^ area indicates the concentration change of the epoxy groups. Sometimes, in order to compare them independently, the absorption bands of the non-reactive group’s main component are used [28,47]. Similarly to [48,49], for the normalization of the 1508 cm^−1^ absorption band, the stretching vibrations of the C=C aromatic bond of the ER were chosen. The results of such processing are shown in Figure 8.

It can be seen that, with an increase of the concentration of DAS, the number of silane groups in the epoxyorganosilane systems grows (Figure 8, curve 1). At the same time, the epoxy group’s content decreases, and approaches zero for the system at 30 wt% (Figure 8, curves 2). Hence, at the DAS concentration of 30–35 wt%, a complete conversion (ω_cc_) of the epoxy groups is achieved.

**Figure 9:**
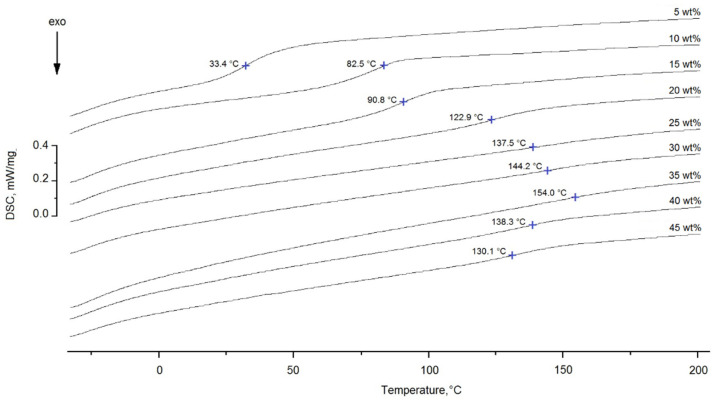
The DSC thermograms of the epoxyorganosilane systems cured at 200 °C.

**Figure 10:**
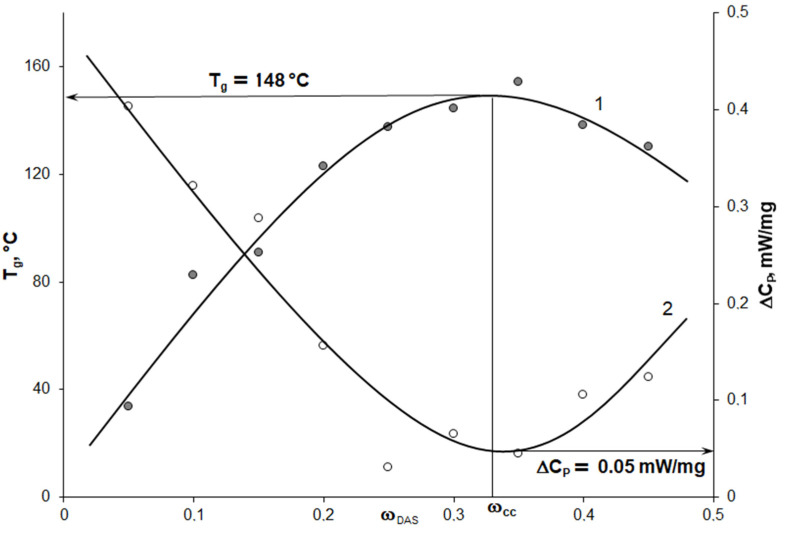
Concentration dependences of the glass transition temperature (1) and of the heat capacity change at T_g_ (2).

The results of the FTIR analysis correlate with the DSC measurements (Figure 9 and Figure 10). As a result of the denser network formation with an increase of the DAS content in the systems from 10 to 30 wt%, the glass transition temperature increases up to 148 °С (Figure 10, curve 1). The formation of a denser network with the increase of the concentration of DAS is also confirmed by the decrease in the change in heat capacity of the system (DC_P_) on passing through the T_g_ (Figure 10, curve 2). For systems with more than 30 wt% DAS concentration, a slight decrease of T_g_ is observed. This confirms the FTIR data on the complete conversion of epoxy groups at 30 wt% DAS, and refers to the plasticization effect of the system.

The surface free energy characteristics of all of the epoxyorganosilane systems after post-curing become close to each other, and are almost similar to the value of the surface free energy (36–38 mJ/m^2^) of the cured epoxy resin. At the same time, the epoxyorganosilane compositions have high values of the dispersive components (30–32 mJ/m^2^) and low values of the polar (5–7 mJ/m^2^) components of surface free energy. This can be explained by the formation of the dense molecular structure of the surface layer.

## 4. Conclusions

It was shown that the epoxyorganosilane systems with different concentrations of DAS (systems 10–45 wt%) in the initial state have low surface free energy characteristics. This makes it possible to use the investigated compositions as a finish for various fibers with higher surface free energy values. The partial curing of the systems at room temperature in an inert atmosphere, followed by contact with air, leads to an increase of the surface free energy of the modified systems up to 75 mJ/m^2^. The variance of the concentration of the initial components allows the adjustment of their surface energy characteristics in the range of 42–75 mJ/m^2^, which makes it possible to use fibers finished with the epoxyaminosilane systems in composite materials based on various thermoset and thermoplastic binders with a surface tension of up to 75 mJ/m^2^.

## Figures and Tables

**Table 1:** The mass and composition proportion of the epoxyorganosilane systems.

Component	The Mass Fractions										
	0 wt%	5 wt%	10 wt%	15 wt%	20 wt%	25 wt%	30 wt%	35 wt%	40 wt%	45 wt%	100 wt%
ER	100	95	90	85	80	75	70	65	60	55	0
DAS	0	5	10	15	20	25	30	35	40	45	100

**Table 2:** Characteristics of the test fluids.

Test Fluids	*ρ*, g/sm^3^	T_boil_, °С	γ^P^_lv_, mJ/m^2^	γ^D^_lv_, mJ/m^2^	γ_lv_, mJ/m^2^
Water	1.00	100.0	50.2	22.0	72.2
Glycerine	1.26	290.0	30.0	34.0	64.0
Formamide	1.1334	210.7	26.0	32.3	58.3
Dimethylsulfoxide	1.096	189.0	8.7	34.9	43.6
o-Tricresilphosphate	1.165	263.0	1.7	39.2	40.9

**Table 3:** Surface free energy characteristics of ER, DAS and hydrolysed DAS.

Used Components	γ^P^, mJ/m^2^	γ^D^, mJ/m^2^	γ, mJ/m^2^
ER	25.4	30.3	55.7
DAS	0.1	35.0	35.1
Hydrolysed DAS	69.4	7.6	77.0

**Table 4:** Surface free energy characteristics of the test surfaces.

Test Surfaces	γ^P^, mJ/m^2^	γ^D^, mJ/m^2^	γ, mJ/m^2^
Polytetrafluoroethylene	0.5	18.6	19.1
Polyethylene	1.1	31.1	32.2
Polyamidoimide	8.4	36.5	44.9

## Data Availability

Not applicable.
